# Differences in Context-Specific Sedentary Behaviors According to Weight Status in Adolescents, Adults and Seniors: A Compositional Data Analysis

**DOI:** 10.3390/ijerph15091916

**Published:** 2018-09-03

**Authors:** Sofie Compernolle, Delfien Van Dyck, Katrien De Cocker, Javier Palarea-Albaladejo, Ilse De Bourdeaudhuij, Greet Cardon, Sebastien F. M. Chastin

**Affiliations:** 1 Department of Movement and Sport Sciences, Faculty of Medicine and Health Sciences, Ghent University, 9000 Ghent, Belgium; delfien.vandyck@ugent.be (D.V.D.); ilse.debourdeaudhuij@ugent.be (I.D.B.); greet.cardon@ugent.be (G.C.); sebastien.chastin@gcu.ac.uk (S.F.M.C.); 2 Research Foundation Flanders (FWO), 1000 Brussels, Belgium; 3 Physically Active Lifestyles Research Group, Institute for Resilient Regions, University of Southern Queensland, Springfield, QLD 4300, Australia; Katrien.Decocker@usq.edu.au; 4 Biomathematics and Statistics Scotland, Edinburgh EH9, UK; javier.palarea@bioss.ac.uk; 5 School of Health and Life Science, Institute of Applied Health Research, Glasgow Caledonian University, Glasgow G12, UK

**Keywords:** domain-specific sedentary behavior, sitting, BMI, compositional data analysis

## Abstract

To develop effective sedentary behavior interventions aimed at people who are overweight/obese, detailed insight is needed into the contexts of sedentary behavior of these people. Therefore, the aims of this study were to describe the composition of sedentary behavior and to compare context-specific sedentary behaviors between different weight groups. Cross-sectional data were used from a study conducted in 2013–2014 among a Flemish sample of adolescents (*n* = 513), adults (*n* = 301), and seniors (*n* = 258). Sixteen context-specific sedentary behaviors were assessed using a validated questionnaire during the week and weekend. Compositional descriptive statistics were performed to determine the relative contribution of context-specific sedentary behaviors in the three age groups. Compositional multivariate analysis of covariance and pairwise comparisons were conducted to examine weight group differences in context-specific sedentary behaviors. The compositional means indicated that the highest proportion of sedentary time was spent at school, at work, and while watching television. Statistically significant differences were found in the composition of sedentary behaviors between healthy weight and overweight/obese participants. In all age groups, socially engaging sedentary behaviors were more prevalent in healthy weight people, whereas socially disengaging behaviors were more prevalent in overweight/obese people. Consequently, the findings of this study suggest that future overweight/obesity interventions should no longer focus on total sedentary time, as not all context-specific sedentary behaviors are associated with overweight/obesity. Instead, it might be better to target specific contexts of sedentary behaviors—preferably those less socially engaging—when aiming to reduce overweight/obesity.

## 1. Introduction

The prevalence of people who are overweight and obesity continues to rapidly increase [1]. Current estimates indicate that about two billion people in the world are overweight or obese [2]. These high prevalence rates are also found in Belgium. Recent studies showed that about 15% of adolescents are overweight [3,4], and more than half of the adults and seniors are overweight [3,5]. This poses a major public health challenge, as being overweight/obese is associated with an increased risk of cardiovascular disease and mortality [6,7,8]. As a result, the demand for health care will rise dramatically, and will present an economic burden on society. A recent study, conducted by Tremmel et al., forecasted that the economic demands associated with being overweight and obese will double between 2010 and 2050 [9]. Consequently, the prevention of becoming overweight and obese has become a main priority in the field of public health.

A promising obesity prevention strategy that has received growing attention in latest years is the reduction of sedentary behavior, as this behavior have been shown to be associated with higher rates of obesity [10,11]. Sedentary behavior (i.e., any waking activity characterized by an energy expenditure ≤1.5 metabolic equivalents (METs) performed in a sitting, reclining, or lying posture [12]) has become ubiquitous in modern societies. A study performed in 2200 European adolescents found a mean—objectively measured—sedentary time of 9 h/day [13]. Pooled accelerometer data from adults of four different European countries [14] revealed an average sedentary time of 8.83 h/day [14], and a recent review on accelerometer-measured sedentary time in seniors showed a daily mean of 9.4 h [15]. As such, it can be concluded that more than half of people’s waking time is spent sedentary.

Although there are different types of sedentary behaviors that occur in a variety of contexts for different purposes, including leisure, household, occupation, and transportation [16,17], existing obesity prevention approaches have often focused on total sedentary time. However, reducing total sedentary time might not be the most appropriate approach to tackle the obesity epidemic as there is emerging evidence that certain (combinations of) sedentary activities may be more harmful for health compared to others [10,18,19]. For example, it has been hypothesized that television and screen time are more detrimental compared to other sedentary behaviors in all age groups [20,21,22] due to the mediating role of unhealthy dietary patterns, lower levels of physical activity, and reduced social networks within this association [23,24,25,26,27]. Moreover, the review of McCormack at al. also showed evidence for a positive association between transport-related sitting time and weight status, as eight out of ten included studies indicated that a higher motor vehicle travel time was associated with a higher BMI [28]. Important to note is that the review of McCormack only included studies with adults. A recent study conducted in adolescents showed no significant association between passive transport and BMI. However, a significant positive association was found between the use of public transport and BMI [29]. In contrast to previously described context-specific sedentary behaviors, the association between occupational sedentary behavior and overweight/obesity remains less clear. A recent review conducted by Shresta et al. showed that nearly half of the existing studies examining the association between occupational sedentary behavior and BMI found no significant association [30]. Moreover, most of the remaining studies reported mixed or unclear results [30]. Furthermore, no evidence is available to date on the negative weight effects of other sedentary behaviors, such as sitting for socializing, for meals, or for reading.

Insight into what sedentary behaviors contribute for overweight/obesity is limited, but highly important in order to develop effective health promotion interventions. Therefore, this study aimed (1) to describe the composition of sedentary behavior in adolescents, adults and seniors and (2) to explore the context-specific sedentary behaviors that are associated with overweight and obesity. This, in order to have a first indication of which (combinations of) sedentary behaviors should be targeted by obesity prevention and management interventions.

## 2. Materials and Methods

### 2.1. Study Design

This study used cross-sectional survey data, collected between April 2013 and May 2014 as part of the Busschaert study. The Busschaert study was designed to determine correlates of sedentary behaviors in adolescents (12–18 years) [31], adults (25–60 years) [32] and seniors (>65 years) [33]. Details on the study protocol, which was approved by the Ethics Committee of Ghent University Hospital (B670201317406), have been published elsewhere [31,32,33].

### 2.2. Recruitment and Participants

Different recruitment strategies were used for the three age groups. To recruit adolescents, sixteen secondary schools in Flanders were contacted via mail or telephone. Of these, seven agreed to participate (response rate: 44%). Headmasters of the participating schools selected classes of different age groups. This resulted in a total of 566 eligible adolescents. Adolescents who agreed to participate in the present study completed a paper-based questionnaire during class time at school. Fifty-three adolescents were not included in the analyses, due to not being present at the moment when filling out the questionnaire (*n* = 29), the lack of permission provided from parents/care givers (*n* = 18) or incomplete data (*n* = 6). This resulted in a final sample of 513 adolescents (participation rate (PR): 90.6%). Adults and seniors were randomly selected by the public service of Sint-Niklaas (i.e., city in Flanders, Belgium, 75,000 inhabitants, 83.8 km^2^). A total of 1917 adults and 961 seniors were invited to participate. Adults were contacted by regular mail to fill out a paper-based questionnaire, and seniors were contacted by telephone to complete a structured questionnaire interview at home. In total, 331 adults filled out the paper-based questionnaire (response rate: 17.5%), and 293 seniors agreed to participate in a face-to-face interview at home (response rate: 30.5%). Of these, 30 adults and 35 seniors were excluded because: their partner filled out the questionnaire (*n* = 21), they were not able to stand (*n* = 8), they were not able to participate due to illness (*n* = 30), they did not speak Dutch (*n* = 4), or they returned the questionnaire after the deadline was exceeded (*n* = 2). This resulted in a final sample of 301 adults (PR: 15.8%) and 258 seniors (PR: 28.1%).

### 2.3. Measures

The questionnaire used in this study was developed by Busschaert et al. and contained questions on sociodemographic characteristics, body weight and height, context-specific sedentary behaviors, and potential social-cognitive, physical environmental and health-related correlates of these behaviors [34]. Only questionnaire items that were used in the present study are explained below.

#### 2.3.1. Context-Specific Sedentary Behaviors

Context-specific sedentary behaviors were assessed using the following question: ‘During the last 7 days, how much time did you usually spend sitting while (1) reading, (2) caring, (3) practicing hobbies, (4) socializing, (5) listening to music, (6) consuming meals, (7) watching television, (8) using a computer for leisure, (9) gaming (only adolescents), (10) taking an afternoon nap (only seniors), (11) moving from one place to another during leisure time, (12) commuting (only adolescents and adults), (13) doing household activities (14) being at work (only adults), (15) doing schoolwork at home (only adolescents), and ,16) being at school (only adolescents). All items were asked separately for week and weekend days, and showed acceptable test-retest reliability (pooled *r* = 0.63). The answer categories slightly differed according to sedentary behaviors, but they all included ordinal scale options ranging from ‘never’ to ‘more than seven hours/day’ [34]. In order to prevent that simultaneous sedentary behaviors are reported twice, participants were instructed to report only the main sedentary behavior (e.g., if one listens to the radio while reading a book, only reading was reported).

#### 2.3.2. Sociodemographic Characteristics and Body Mass Index 

Sociodemographic characteristics include age, gender, family situation (single; having a partner, but living independently; living with a partner; being married; widow/widower), educational level (high (i.e., completed tertiary education); low (i.e., did not completed tertiary education)), having children (yes; no), occupational status (working full-time; working part-time; household; unemployed/job-applicant; career interruption; retired; student), and type of education (vocational secondary education; technical secondary education; general technical education). Family situation, educational level, and having children were only asked in adults and seniors. Occupational status was only asked in adults, and type of education was only asked in adolescents. Body mass index was calculated by dividing weight in kilograms by the square of height in meters. Adolescents’ and adults’ weight and height were asked in the questionnaire and seniors’ weight and height were objectively measured by a trained researcher using a SECA 813 Robusta weight scale, and a SECA 213 portable stadiometer, respectively. Adolescents were categorized in healthy weight and overweight/obese according to their BMI, using the cut-off points for age and gender defined by Cole and colleagues [35]. Adults and seniors with a BMI value ≥18.5 kg/m^2^ and <25 kg/m^2^ were categorized as healthy weight, whereas adults and seniors with a BMI value ≥25 kg/m^2^ were categorized as overweight/obese. Underweight participants (i.e., BMI < 18.5 kg/m²) were excluded from the analyses.

### 2.4. Statistical Analyses

The included context-specific sedentary behaviors data provide only relative information, as they all represent parts of total sedentary behavior (i.e., 100%). If the proportion of time devoted to one context-specific sedentary behavior changes, this will affect the relative values of at least one of the other sedentary behaviors. As such, context-specific sedentary behaviors are codependent, and should thus be handled as compositional data [36]. Standard descriptive statistics do not account for this compositional structure of the data (i.e., all context-specific sedentary behaviors are fractions of a total time of observation); therefore, compositional descriptive statistics were used instead. As a measure of central tendency, the geometric mean for each context-specific sedentary behavior was calculated and normalized to 100% to obtain the so-called compositional mean. The total or metric variance was calculated as a measure of overall dispersion of the sedentary behavior compositions [37]. Furthermore, following the methodological developments in [37,38], log-ratio transformations were performed on the original compositional data to map them into equivalent real-valued coordinates. These transformations thus facilitated the application of conventional statistical methods generally used on non-compositional data. Firstly, two compositional multivariate analysis of covariance (MANCOVA) models were fitted in log-ratio coordinates per age group (i.e., one for weekday sedentary behavior and another one for weekend day sedentary behavior) to test if the mean composition of sedentary behaviors statistically significantly differed between the healthy weight group and the overweight/obese group after controlling for gender and educational level. Subsequently, pairwise group comparisons were conducted by computing and graphically representing 95% bootstrap percentile confidence intervals of log-ratio differences between group means. This allowed investigating which behaviors were responsible for the difference in mean composition. Statistical test significance was assessed at the usual 5% significance level. The statistical analyses were conducted on the R system for statistical computing v3.2 (R Core Team 2016, R Foundation for Statistical Computing, Vienna, Austria, http://www.r-project.org).

## 3. Results

### 3.1. Sample Characteristics

The study sample consisted of 1072 participants; 513 adolescents (mean age: 15.0 ± 1.7), 301 adults (mean age: 43.3 ± 24.6 years) and 258 seniors (mean age: 74.0 ± 6.2 years). Demographic characteristics of the study sample are presented in Table 1.

### 3.2. Descriptive Statistics of Week and Weekend Day Context-Specific Sedentary Behaviors

Table 2 and Table 3 shows the descriptive statistics of the proportion of time spent in context-specific sedentary behaviors obtained via compositional statistics. Compositional means and total variances were presented separately for week and weekend day sedentary behaviors in healthy weight and overweight/obese adolescents, adults and seniors. For adolescents, the compositional means indicated that the highest proportion of weekday sedentary time was spent at school (42.95%), followed by watching television (14.11%) and using a computer (9.72%). For adults, the highest proportion of weekday sedentary time was spent at work (30.02%), closely followed by watching television (28.38%) and consuming meals (12.53%). For seniors, the highest proportion of weekday sedentary time was spent while watching television (47.88%), consuming meals (22.86%) and reading (10.77). The highest proportion of weekend day sitting time was spent while watching television for all age-groups (i.e., 27.92% for adolescents, 36.79 for adults and 44.51% for seniors). The top three of weekend day sedentary behaviors was completed by using a computer (16.17%) and consuming meals (11.12%) in adolescents, by consuming meals (18.08%) and socializing (11.91%) in adults, and by consuming meals (27.74%) and reading (10.32%) in seniors. The obtained total variances indicated that the healthy weight group was generally more homogenous (i.e., lower total variability) compared to the overweight/obese group in overall sedentary behavior across all age groups.

### 3.3. Weight Group Differences in Context-Specific Sedentary Behavior

**Figure 1:**
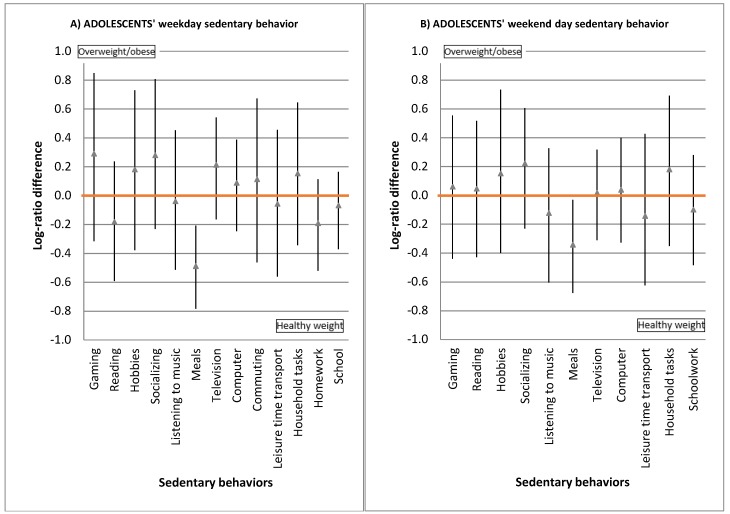

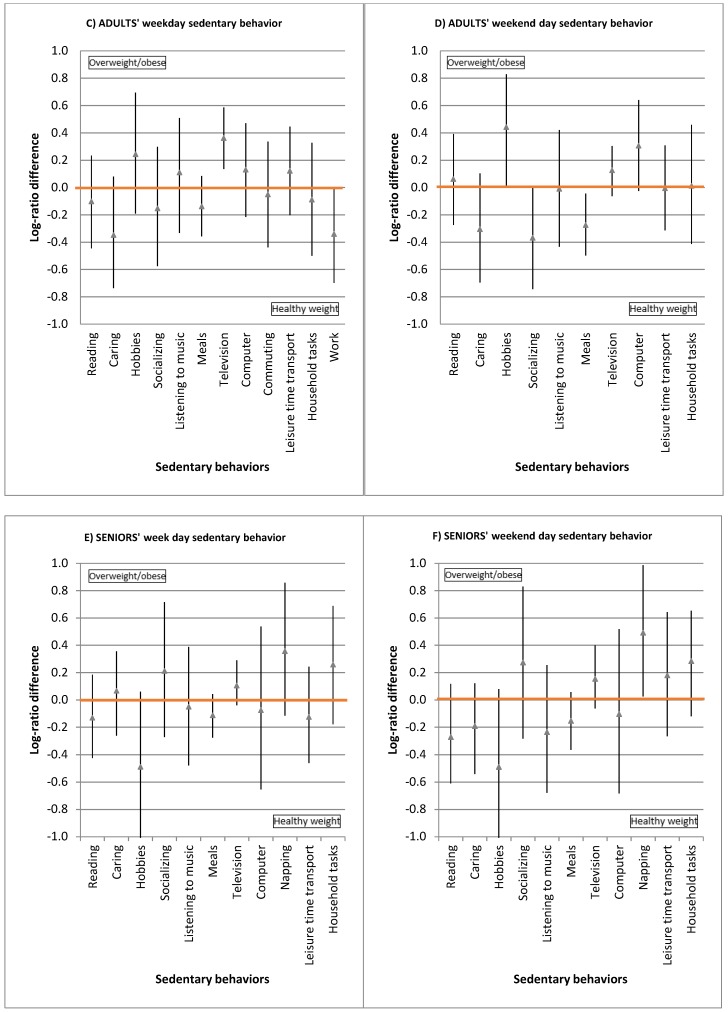
95% bootstrap percentile confidence intervals for log-ratio differences in context-specific sedentary behaviors between compositional means of weight groups in adolescents (**A**,**B**), adults (**C**,**D**), and seniors (**E**,**F**). The triangles represent the estimated log-ratio difference between the compositional means for each sedentary behavior, and the vertical lines are the associated 95% bootstrap percentile confidence intervals. Intervals not including the value zero reflect on relevant differences between weight groups Intervals ranging in the positive side indicate behaviors that are more prevalent among overweight/obese people compared to healthy weight people. Conversely, intervals ranging in the negative side indicate the opposite.

Results from the MANCOVA test revealed statistically significant differences in the mean composition of sedentary behaviors between weight groups in adolescents (*p* = 0.01), adults (*p* < 0.001) and seniors (*p* = 0.01). Figure 1 displays pairwise comparisons between weight groups. The triangles represent the estimated log-ratio difference between the compositional means for each sedentary behavior, and the vertical lines are the associated 95% bootstrap percentile confidence intervals. Intervals not including the value zero reflect on relevant differences between weight groups. Thus, our results indicate that—both at week and weekend days—a significant higher proportion of sedentary time was spent for meals in healthy weight adolescents compared to overweight/obese adolescents.

Compared to the overweight and obese adults, healthy weight adults reported significant higher proportions of sedentary time at work (weekday), for socializing (weekend day) and for meals (weekend day), whereas overweight and obese adults reported significant higher proportions of sedentary time while watching television (weekday) and for hobbies (weekend day) compared to healthy weight adults. A significant higher proportion of sedentary time was spent for napping (weekend) in overweight and obese seniors, compared to their healthy weight counterparts.

## 4. Discussion

The present study aimed to describe the composition of context-specific sedentary behaviors in adolescents, adults and seniors and to identify context-specific sedentary behaviors that are associated with healthy weight and overweight/obese groups. To date, no previous study has tried to break down sedentary time into a comprehensive set of distinct behaviors in relation to overweight and obesity. However, examining the association with overweight and obesity is important to determine the sedentary behaviors with the greatest potential for future obesity prevention interventions.

In general, our results support the hypothesis that some sedentary behaviors are more prevalent among overweight/obese participants compared to others. More specifically, our results indicate that sitting for watching television, for hobbies and for napping are more prevalent in overweight and obese people, whereas sitting for meals, for socializing, at school and at work are more prevalent in healthy weight people. As such, it seems that socially disengaging sedentary behaviors are predominant among overweight/obese people, whereas socially engaging sedentary behaviors are predominant among healthy weight people.

Although the underlying mechanisms for these associations were not examined in this study, our results may suggest that the associations were mediated by a social element, such as social networks or loneliness. Previous literature has shown that loneliness is associated with obesity [39]—and thus it might be the case that people who spend a lot of time in socially disengaging sedentary behavior are more lonely, and thus at higher risk for obesity. Next to the potential underlying mechanisms of loneliness, it might be worth studying the mediating effects of physical activity and dietary behavior. Previous literature has indicated that television is more strongly related to unhealthy dietary intake [25,40], and lower levels of physical activity [41] compared to other sedentary behaviors. As such, these other energy-balance related behaviors might also explain why certain sedentary behaviors are more prevalent among overweight and obese participants compared to others.

If the causality of the current associations are confirmed by future longitudinal studies, socially disengaging sedentary behavior—such as sitting for watching television, for hobbies and for napping—should be the main target of future overweight/obesity prevention approaches. A first strategy might be to encourage people to reduce their socially disengaging sedentary behavior. However, this might be challenging as many people engage in these behaviors because they find it enjoyable, entertaining [31,32], and because it makes them feel revitalized [42]. Alternative strategies might be to encourage people to engage in these behaviors while standing—by for example using a standing table, or to stimulate adults to break up prolonged periods of sitting time while watching television/practicing hobbies. By doing so, the metabolic changes associated with prolonged sitting will be inhibited [43], and negative health effects will diminish [43,44]. This way, people can continue to carry out their usual activities in a healthy manner. The latter two strategies might be feasible options to watch television, and to practice hobbies, but impossible for napping. However, as previous research seem to indicate that napping has the potential to bring undeniable benefits [45], such as better well-being, improved sleep quality, and enhanced cognitive performance, one must be careful when targeting this context in future sedentary behavior interventions.

A main strength of this study is the application of compositional methods to a unique dataset including sixteen different contexts of sedentary behavior in three diverse age groups. By applying this innovative approach, analyses were adjusted for the interdependence between times spent on different sedentary behaviors. Furthermore, context-specific sedentary behaviors were assessed using reliable and valid questionnaires. Moreover, the older adult questionnaires were conducted through face-to-face interviews, as seniors may experience cognitive difficulties when responding to a paper-based questionnaire [18]. In this way, more precise answers can be obtained as the interviewer is able to provide additional information if necessary. Important study limitations include the cross-sectional nature of the data. This comprises that age-group differences could be confounded by cohort effects, and that causality of the findings cannot be established. Longitudinal studies would be useful to disentangle whether certain compositions of sedentary behavior result in overweight/obesity or if it is the other way around (i.e., that overweight and obese people are more likely to engage in certain compositions of sedentary behavior). Secondly, the response rate in adults was rather low, which might have led to selection bias. Although there is a good representation of men and women, lower and higher educated adults as well as of different age groups in the study, it remains plausible that those who are more concerned with their health or those more motivated to limit their sitting time be physically active were more likely to have completed the questionnaire [36]. Finally, the use of self-reported questionnaires to assess context-specific sedentary behaviors and BMI may have resulted in measurement error due to for example social desirability and recall biases. It has been shown that respondents tend to under-report their sedentary behaviors when completing questionnaires. By using global positioning systems or wearable cameras next to accelerometers, context-specific sedentary time could be determined objectively in future research [8].

## 5. Conclusions

In conclusion, our findings support the idea that not all context-specific sedentary behaviors are equally related to weight status; this implies that future obesity prevention and management interventions should focus on decreasing the specific sedentary behaviors that seem to be more obesogenic rather than on the indiscriminate reduction of total sedentary time. More specifically, our results suggest that overweight and obese people spend a higher proportion of their sedentary time in socially disengaging sedentary behaviors, such as sitting while watching television, while napping, and for hobbies. Therefore—if confirmed by future longitudinal studies—these behaviors should be targeted in sedentary behavior interventions aimed at the reduction of overweight and obesity.

## Figures and Tables

**Table 1:** Sociodemographic characteristics of the participants.

Sociodemographic Variables and BMI	Adolescents (*n* = 513)	Adults (*n* = 301)	Seniors (*n* = 258)
**Age**: years, mean (SD)	15.0 (1.7)	43.3 (24.6)	74.0 (6.2)
**Gender**: %			
Male	64.3	45.5	47.3
**Family situation**: %			
Single	-	12.2	11.7
Partner but living apart	-	7.0	2.3
Married/living with partner	-	79.0	67.7
Widow/widower	-	1.7	18.3
**Type of education**: %			
Vocational secondary education	13.9	-	-
Technical secondary education	52.7	-	-
General secondary education	33.3	-	-
**High educational level** *: %	-	52.2	24.3
**Occupational status**: %			
Full-time job	-	71.9	-
Part-time job	-	17.1	-
Household	-	5.4	-
Unemployed/job-applicant	-	2.7	-
Career interruption	-	1.0	-
Retired	-	1.0	-
Student	-	1.0	-
**Having children**: %			
Yes	-	71.6	90.3
**Body mass index**: kg/m², mean (SD)	19.82 (2.96)	24.6 (3.5)	27.8 (4.0)
Healthy weight (%)	85.6	63.3	25.9

* Completed college or university.

**Table 2:** Compositional descriptive statistics of the percentage of time spent in weekday context-specific sedentary behaviors by age and weight group.

Context-Specific Sedentary Behaviors	Adolescents			Adults			Seniors		
	Total	Healthy Weight	Overweight/Obese	Total	Healthy Weight	Overweight/ Obese	Total	Healthy Weight	Overweight/Obese
WEEKDAY									
Gaming (%)	3.52	3.37	4.50	-	-	-	-	-	-
Reading (%)	0.80	0.82	0.69	4.51	4.62	4.23	10.77	11.86	10.39
Caring (%)	-	-	-	1.03	1.16	0.82	0.44	0.42	0.44
Hobbies (%)	0.61	0.59	0.72	1.03	0.94	1.19	2.76	4.05	2.41
Socializing (%)	2.78	2.66	3.55	2.32	2.43	2.09	4.49	3.87	4.72
Music (%)	3.62	3.63	3.50	1.36	1.30	1.44	0.76	0.79	0.75
Meals (%)	7.45	7.97	4.91	12.53	13.04	11.40	22.86	24.90	22.11
TV (%)	14.11	13.63	17.05	28.38	24.60	35.43	47.88	44.15	49.11
PC (%)	9.72	9.57	10.53	7.92	7.46	8.57	2.43	2.55	2.39
Afternoon nap (%)	-	-	-	-	-	-	1.40	1.07	1.54
Commuting (%)	1.70	1.68	1.87	3.78	3.81	3.63	-	-	-
Leisure time transport (%)	2.31	2.32	2.18	4.02	3.81	4.32	4.44	4.88	4.28
Household/telephone (%)	1.77	1.73	2.03	3.08	3.16	2.88	1.76	1.48	1.87
Work (%)	-	-	-	30.02	33.68	23.99	-	-	-
Schoolwork (%)	8.65	8.86	7.41	-	-	-	-	-	-
School (%)	42.95	43.17	41.06	-	-	-	-	-	-
Total variance	31.41	30.97	34.00	24.94	24.11	26.30	22.67	21.38	23.09

Note: The percentages reflect the proportion of total sedentary time that is spent in one specific context per age and weight group. The sum of each column is 100%, reflecting total sedentary behavior.

**Table 3:** Compositional descriptive statistics of the percentage of time spent in weekend day context-specific sedentary behaviors by age and weight group.

Context-Specific Sedentary Behaviors	Adolescents			Adults			Seniors		
	Total	Healthy Weight	Overweight/Obese	Total	Healthy Weight	Overweight/Obese	Total	Healthy Weight	Overweight/Obese
WEEKEND DAY									
Gaming (%)	9.45	9.34	10.04	-	-	-	-	-	-
Reading (%)	1.49	1.48	1.55	7.04	6.82	7.33	10.32	12.65	9.56
Caring (%)	-	-	-	1.20	1.33	0.98	0.45	0.52	0.42
Hobbies (%)	1.12	1.09	1.28	1.50	1.27	1.97	2.01	2.94	1.76
Socializing (%)	7.81	7.54	9.57	11.91	13.57	9.38	5.27	4.35	5.60
Music (%)	6.29	6.39	5.68	1.73	1.73	1.70	0.78	0.93	0.73
Meals (%)	11.12	11.65	8.34	18.08	19.82	15.18	27.74	31.17	26.49
TV (%)	27.92	27.74	28.75	36.79	34.90	39.69	44.51	39.51	46.15
PC (%)	16.17	16.03	16.91	9.73	8.62	11.80	2.31	2.49	2.24
Afternoon nap (%)	-	-	-	-	-	-	1.44	0.99	1.63
Commuting (%)	-	-	-	-	-	-	-	-	-
Leisure time transport (%)	3.89	3.96	3.51	7.75	7.75	7.75	3.72	3.27	3.88
Household/telephone (%)	2.73	2.65	3.24	4.21	4.19	4.21	1.45	1.19	1.54
Work (%)	-	-	-	-	-	-	-	-	-
Schoolwork (%)	12.01	12.14	11.12	-	-	-	-	-	-
School (%)	-	-	-	-	-	-	-	-	-
Total variance	28.70	28.44	30.51	20.58	19.85	21.66	25.02	24.09	25.24

Note: The percentages reflect the proportion of total sedentary time that is spent in one specific context per age and weight group. The sum of each column is 100%, reflecting total sedentary behavior.
